# Emergence of 16S rRNA methylase-producing *Acinetobacter baumannii* and *Pseudomonas aeruginosa* isolates in hospitals in Vietnam

**DOI:** 10.1186/1471-2334-13-251

**Published:** 2013-05-30

**Authors:** Tatsuya Tada, Tohru Miyoshi-Akiyama, Yasuyuki Kato, Norio Ohmagari, Nozomi Takeshita, Nguyen Viet Hung, Doan Mai Phuong, Truong Anh Thu, Nguyen Gia Binh, Nguyen Quoc Anh, Tran Thi Thanh Nga, Pham Hong Truong, Phan Thi Xuan, Le Thi Anh Thu, Nguyen Truong Son, Teruo Kirikae

**Affiliations:** 1 Department of Infectious Diseases, National Center for Global Health and Medicine, Shinjuku, Tokyo 162-8655, Japan; 2 Disease Control and Prevention Center, National Center for Global Health and Medicine, Shinjuku, Japan; 3 Disease Control and Prevention Center, Division of Infectious Diseases, National Center for Global Health and Medicine, Shinjuku, Japan; 4 Bach Mai Hospital, Hanoi, Vietnam; 5 Cho Ray Hospital, Ho Chi Minh, Vietnam

**Keywords:** *Acinetobacter baumannii*, *Pseudomonas aeruginosa*, Aminoglycoside resistance, Intensive care unit, 16S rRNA methylase

## Abstract

**Background:**

16S rRNA methylase-producing Gram-negative bacteria are highly resistant to all clinically important aminoglycosides. We analyzed clinical strains of 16S rRNA methylase-producing *Acinetobactor baumannii* and *Pseudomonas aeruginosa* obtained from clinical isolates in medical settings in Vietnam.

**Methods:**

From 2008 to 2011, 101 clinical strains of *A. baumannii* and 15 of *P. aeruginosa* were isolated from patients in intensive care units (ICUs) in two medical settings in Vietnam. Antimicrobial susceptibilities were determined using the microdilution method and epidemiological analysis was performed by pulsed-field gel electrophoresis and MLST. Genes encoding the 16S rRNA methylases, OXAs and CTX-Ms were analyzed by PCR and sequence analysis.

**Results:**

16S rRNA methylase-producing Gram-negative pathogens were detected in two hospitals in Vietnam. Of the 101 clinical isolates of *A. baumannii* and the 15 of *P. aeruginosa* isolated from two ICUs in these hospitals, 72 (71.3%) were highly resistant to amikacin, arbekacin and gentamicin, with MICs greater than 1,024 mg/L. The 16S rRNA methylases ArmA and RmtB were produced by 61 and 9 isolates of *A. baumannii*, respectively, and RmtB was produced by 2 isolates of *P. aeruginosa*. Moreover, 52 of the *A. baumannii* isolates producing 16S rRNA methylases harbored both *bla*OXA-23-like and *bla*OXA-51-like genes. Most *A. baumannii* isolates producing 16S rRNA methylase obtained in hospital A in Hanoi were ST91 and ST231, whereas most from hospital B in Ho Chi Minh City were ST136, ST195, and ST254.

The two *P. aeruginosa* isolates harboring *rmtB* showed different patterns on PFGE, one each corresponding to ST217 and ST313.

**Conclusions:**

Gram-negative bacteria producing the 16S rRNA methylases ArmA and RmtB are emerging in medical settings in Vietnam. *A. baumannii* isolates in northern and southern regions of Vietnam may be of different lineages.

## Background

Aminoglycosides widely used to treat infectious diseases caused by Gram-negative bacteria have a high affinity for the 16S rRNA of the bacterial 30S ribosome and block protein synthesis [1]. Enzymatic modification [1] and the methylation of 16S rRNA makes these bacteria highly resistant to all clinically important aminoglycosides [2]. In 2003, clinical isolates of highly aminoglycoside-resistant Gram-negative bacteria producing16S rRNA methylase were identified in France [3] and Japan [4]. Since then, 16S rRNA methylase-producing Gram-negative bacteria have been isolated in other parts of the world, including Asian countries such as Afghanistan, Bangladesh, China, Hong Kong, India, Japan, Korea, Oman and Pakistan [5]. To date, however, there have been no reports of these isolates from South-East Asian countries, including Vietnam.

Since 2003, eight plasmid-associated 16S rRNA methylase genes, *armA, rmtA, rmtB, rmtC, rmtD, rmtE, rmtF* and *npmA*, have been identified in clinical and veterinary isolates from various geographic areas, including East Asia, Europe and the Americas, since 2003 [5,6].

## Methods

### Bacterial strains

From 2008 to 2011, 50 clinical strains of *A. baumannii* and 15 of *P. aeruginosa* were isolated from patients in an ICU in hospital A in Hanoi, Vietnam; and 51 strains of *A. baumannii* were isolated from patients in an ICU in hospital B in Ho Chi Minh City, Vietnam. Of the 101 *A. baumannii* strains isolated, 98 were from patients’ respiratory tracts and 3 from blood. Of the 15 *P. aeruginosa* strains, 14 were from respiratory tracts and 1 from pus. Most patients were on ventilators, and the samples were mostly aspirates from ventilation tubes. All clinical isolates used in this study were obtained during standard patient care.

### Antimicrobial susceptibility and pulsed-field gel electrophoresis (PFGE)

MICs of all bacteria to amikacin (Sigma-Aldrich, St. Louis, MO), arbekacin (Meiji Seika Pharma Co., Tokyo, Japan), aztreonam (Eizai, Tokyo, Japan), ceftadizime (Sigma-Aldrich), ciprofloxacin (Daiichi Pharmaceutical Co, Tokyo, Japan), colistin (Sigma-Aldrich), gentamicin (Nacalai Tesque, Kyoto, Japan), imipenem (Banyu Pharmaceutical Co, Tokyo, Japan), meropenem (Sumitomo Pharmaceutical Co., Osaka, Japan), piperacillin (Sigma-Aldrich) and pipiracillin/tazobactam (Toyama Chemical Co., Tokyo, Japan) were determined using the microdilution method, according to the guidelines of the Clinical and Laboratory Standards Institute (M07-A9). *A. baumannii* DNA was digested with the restriction enzyme *ApaI* and *P. aeruginosa* DNA was digested with *SpeI*, followed by pulsed-field gel electrophoresis (PFGE). PFGE analysis was performed as described previously [7]. Fingerprinting patterns were analyzed by the unweighted-pair-group method using Molecular Analyst Fingerprinting Plus software (Bio-Rad Laboratories, Hercules, CA, USA) to create an average linkage-based dendrogram.

### Multilocus sequence typing (MLST)

MLST of 16S rRNA methylase-producing pathogens was performed according to the protocols described on the *A. baumannii* (http://pubmlst.org/abaumannii/) and *P. aeruginosa* (http://pubmlst.org/paeruginosa/) MLST Database websites. Seven chromosomal genes were PCR amplified and sequenced, with their nucleotide sequences compared with the sequences submitted to the MLST database to determine allele numbers and STs.

### Detection of aminoglycoside-resistant genes

PCR with 16S rRNA methylase gene specific primers [2,8,9] was performed to detect the *armA, rmtA, rmtB, rmtC, rmtD, rmt E* and *npmA* genes. All PCR amplicons were sequenced using an ABI PRISM 3130 sequencer (Applied Biosystems, Foster City, CA, USA).

Whole genomes of methylase-negative *A. baumannii* and *P. aeruginosa*, which had MICs 128 mg/L to amikacin, 32 mg/L to arbekacin and 128 mg/L to gentamicin, were extracted by DNeasy Blood & Tissue kit (QIAGEN, Tokyo, Japan) and sequenced by MiSeq (Illumina, San Diego, CA). The sequence data were used to confirm aminoglycoside-resistant genes.

### Detection of OXAs and CTX-Ms encoding genes

The presence of *bla*OXA-23-like, *bla*OXA-24-like, *bla*OXA-51-like, *bla*OXA-58-like and *bla*CTX-Ms in 16S rRNA methylase-producing isolates were investigated by PCR [10,11].

### Determination of the genetic environment surrounding *rmtB*

A draft genome sequence of an isolate of *A. baumannii*, NCGM36, harboring *rmtB* was determined using the GS Junior System (Roche Diagnostics K.K, Tokyo).

### Ethical approval

This study was approved in 2007 by Ministry of Health, Bach Mai Hospital (Memorandum of agreement for the collaborative research project on epidemiology of nosocomial infections at the Bach Mai Hospital) and in 2011 by Cho Ray Hospital (approval number: 1644/QD-BVCR), and by the Biosafety Committee, National Center for Global Health and Medicine (approval number: 23-M-49).

## Results

### Antimicrobial susceptibility and aminoglycoside-resistant genes

**Figure 1:**
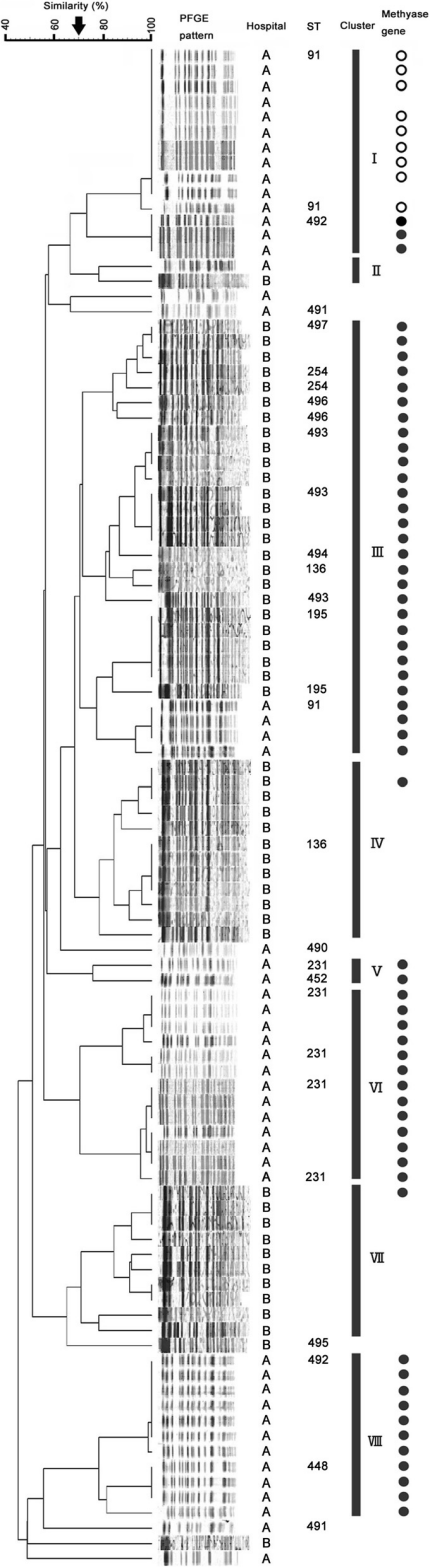
**PFGE pattern and MLST analysis of 101 *Acinetobacter baumannii *isolates.** Eight clusters (I-VIII) with more than 70% similarity were identified. Isolates harboring *armA* (●) and *rmtB* (○) are shown in the column on the right.

The MICs at which 50% and 90% of the 101 *A. baumannii* and 15 *P. aeruginosa* isolates were inhibited (MIC50 and MIC90, respectively) were determined (Table 1). Seventy of the 101 *A. baumannii* isolates (71.3%) had MICs >1,024 mg/L to all aminoglycosides tested, including amikacin, arbekacin and gentamicin. All 70 isolates had 16S rRNA methylases, with 61 having *armA* and the remaining 9 having *rmtB* (Figure 1). The remaining 31 isolates had MICs ≤128 mg/L to amikacin, ≤32 mg/L to arbekacin and ≤128 mg/L to gentamicin and no methylase genes. Whole genome sequencing of 2 methylase-negative isolates showing relative resistance to aminoglycosides revealed that one had *aac(6’)-IIb* and *aadB* and that the other had *aac(6’)-IIb* and *aadA2*.

**Table 1:** **MIC50 and MIC90 values and percent antimicrobial resistance for *A. baumannii *and *P. aeruginosa *clinical isolates**

**Antimicrobial agent**		***A. baumannii *(n = 101)**				***P. aeruginosa *(n = 15)**				
	**Breakpoint forresistance**^***a ***^**(mg/L)**	**%Resistance**	**Range(mg/L)**	**MIC**_**50**_**(mg/L)**	**MIC**_**90**_**(mg/L)**	**Breakpoint forresistance**^***a ***^**(mg/L)**	**%Resistance**	**Range(mg/L)**	**MIC**_**50**_**(mg/L)**	**MIC**_**90**_**(mg/L)**
Amikacin	≥64	85	<2- > 1024	>1024	>1024	≥64	60	<2- > 1024	64	>1024
Arbekacin	-	-	<2- > 1024	>1024	>1024	-	-	2- > 1024	2	>1024
Aztreonam	-	-	<2- > 256	128	>256	≥32	46	4- > 256	16	>256
Ceftazidime	≥32	55	<4- > 512	>512	>512	≥32	46	<4- > 512	16	>512
Ciprofloxacin	≥4	98	<1- > 128	128	>128	≥4	66	<1-64	8	32
Colistin	≥4	6	<0.25-4	2	2	≥8	0	<0.25-0.5	<0.25	0.5
Gentamicin	≥16	91	<2- > 1024	>1024	>1024	≥16	66	<2- > 1024	256	>1024
Imipenem	≥16	48	<4-128	16	32	≥8	53	<4-64	16	32
Meropenem	≥16	51	<4- > 256	32	64	≥8	53	<4-32	16	32
Piperacillin	≥128	100	<4- > 512	>512	>512	≥128	46	<4- > 512	32	256
Piperacillin/Tazobactam	≥128/4	52	<4- > 512	256	512	≥128/4	13	<4-512	32	128

^*a*^Breakpoints for antimicrobial resistance were determined according to the guidelines of the Clinical and Laboratory Standards Institute (M07-A9).

**Figure 2:**
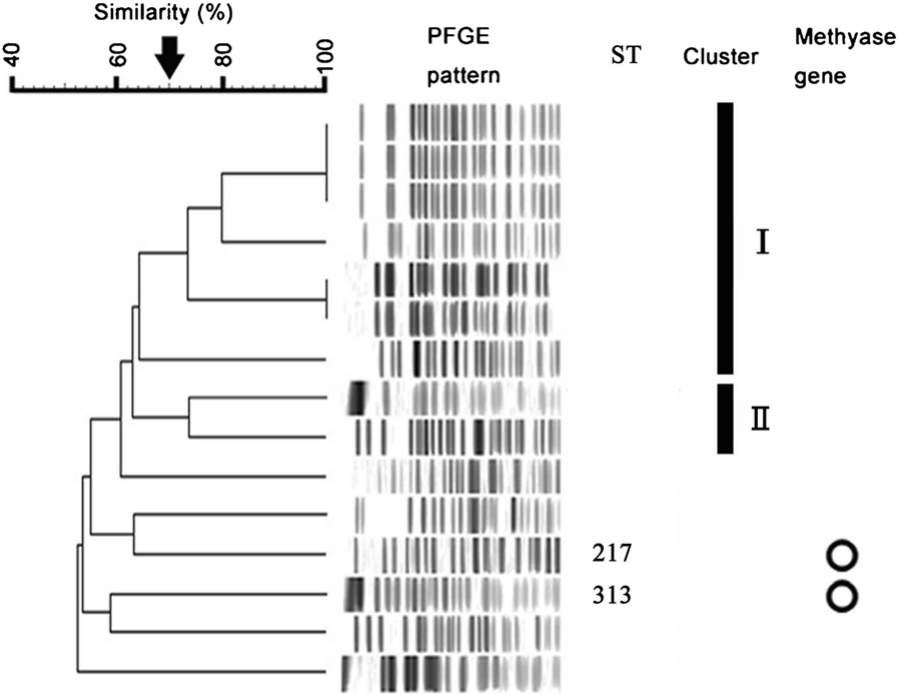
**PFGE pattern and MLST analysis analysis of 15 *Pseudomonas aeruginosa *isolates.** Eight clusters with more than 70% similarity were detected. Two clusters (I and II) with more than 70% similarity were identified. Isolates harboring *rmtB* (○) are shown in the column on the right.

Of the 15 *P. aeruginosa* isolates, 2 had MICs >1,024 mg/L to amikacin, arbekacin and gentamicin, and harbored the 16S rRNA methylase *rmtB* (Figure 2). The 13 methylase-negative isolates had MICs <2 - 256 mg/L to amikacin (MIC_50_ 64 mg/L and MIC_90_ 128 mg/L), 1–32 mg/L to arbekacin (MIC_50_ 2 mg/L and MIC_90_ 4 mg/L), and 1–32 mg/L to <0.5 - 512 mg/L to gentamicin (MIC_50_ 256 mg/L and MIC_90_ 512 mg/L). The remaining 13 did not have any methylase genes (Figure 2).

### OXAs and CTX-Ms encoding genes in 16S rRNA methylase-producing isolates

Of the 61 *A. baumannii* isolates harboring *armA*, 1 had *bla*OXA-23-like, *bla*OXA-51-like and *bla*CTX-Ms genes, 51 had *bla*OXA-23-like and *bla*OXA-51-like genes, and 8 had *bla*OXA-51-like genes. All 9 *A. baumannii* isolates harboring *rmtB* had *bla*OXA-23-like and *bla*OXA-51-like genes. In contrast, the 2 *P. aeruginosa* isolates harboring16S rRNA methylase genes had neither the *bla*OXAs nor the *bla*CTX-Ms gene.

### PFGE analysis and MLST

PFGE analysis of the 101 *A. baumannii* isolates revealed 8 clusters (Figure 1). Isolates from Clusters I, III, IV, V, VI, VII, and VIII were obtained from either one or the other hospital, whereas isolates from Clusters II and III were obtained from both. These results indicate that *A. baumannii* isolates had expanded in a clonal manner in both hospitals and that some isolates may spread among hospitals in Vietnam.

The16S rRNA methylase-encoding the *rmtB* gene was detected in Cluster I *A. baumannii* isolates, whereas *armA* was present in isolates from Clusters I, III, IV, V, VI, VII, and VIII. Isolates harboring *rmtB* were obtained from one hospital and isolates harboring *armA* were from both hospitals.

The *A. baumannii* isolates producing 16S rRNA methylase belonged to ST254, ST231, ST195, ST136, ST91 and 8 new STs, ST490, ST491, ST492, ST493, ST494, ST495, ST496 and ST497 (Figure 1). Most of the *A. baumannii* isolates producing 16S rRNA methylase from hospital A in Hanoi were ST91 and ST231, whereas most from hospital B in Ho Chi Minh City were ST136, ST195 and ST254.

The two isolates harboring *rmtB* showed different patterns on PFGE, belonging to ST217 and ST313.

### Genetic environment surrounding *rmtB*

The *rmtB* gene was associated with an ISCR3 mobile element upstream and a Tn3 transposon structure *bla*TEM-1-*tnpR*-*tnpA* downstream (data not shown). The genetic environment of *rmtB* had more than 99.9% nucleotide sequence identity, from nucleotide 1 to 8,337, to plasmid pXD2 (Gen bank accession no. JN315966) in *E. coli*, which causes bovine milk mastitis in China [12]*.* NCGM36, which harbored *rmtB*, had the *bla*OXA-23 and *bla*OXA-68 genes, but had neither the *aac(6’)-Ib-cr* nor the *bla*CTX-Ms gene.

## Discussion

The high prevalence of 16S rRNA methylase producing Gram-negative bacteria in hospitals in Vietnam may have resulted from the high rate of use of aminoglycosides. It has been estimated that 67.4% of hospitalized patients in Vietnam received antibiotics, including 18.9% who received aminoglycosides, with many 30.8% of these prescriptions considered inappropriate [13]. This rate of antibiotic use was much higher than in European countries (17.8%-32.0%) [14,15]. Moreover, the rate of inappropriate indications for antibiotic prescriptions in hospitals in Vietnam was much higher than rates reported in Malaysia (4.0%) [16], Turkey (14.0%) [17], and Hong Kong (20.0%) [18].

*A. baumannii* isolates from the northern and southern regions of Vietnam may be of different lineages. To date, 2 strains of *A. baumannii* showing ST91 and 3 showing ST136 have been isolated in China; 6 strains showing ST195 have been isolated, 1 in Norway, 2 in Thailand, 2 in Malaysia and 1 in China;, 5 strains showing ST231 have been isolated in Brazil and 1 strain showing ST254 has been isolated in China (http://pubmlst.org/abaumannii/). ST136 and ST195 belong to clonal complex 92, the most widely disseminated complex worldwide [19]. Two strains of *P. aeruginosa* producing RmtB, showing ST217 and ST313, may have originally derived from Europe or Australia, because, to date, *P. aeruginosa* ST217 isolates were obtained only in the United Kingdom and ST313 isolates only in Australia, France and Hungary [20] (http://pubmlst.org/paeruginosa/).

To our knowledge, this is the first report showing that *A. baumannii* strains harboring a 16S rRNA methylase (ArmA or RmtB) and with *bla*OXA-23-like and *bla*OXA-51-like genes are emerging in medical settings in Vietnam. ArmA and OXA-23-like producing Gram-negative pathogens have been reported in Bulgaria [21], France [22], India [23], Korea [24], Norway [25] and the United States of America [26], and ArmA and OXA-51-like producing strains have been reported in Japan [27]. Moreover, *armA* and *rmtB* have been linked to *bla*CTX-Ms [28,29], but almost all ArmA producing isolates in Vietnam did not harbor *bla*CTX-Ms.

We found that some *A. baumannii* clinical isolates harbored *rmtB*. The genetic environment of the *rmtB* regions was very similar to the nucleotide sequence, from nt 1 to nt 8,337, of the plasmid pXD2. However, the plasmid of NCGM36 likely differs from pXD2 (Gen bank accession no. JN315966), in that the former NCGM36 did not have *aac(6’)-Ib* or *bla*CTX-Ms.

Since 16S rRNA methylase genes in *A. baumannii* and *P. aeruginosa* are located in transferable plasmids [5], the absence of methylase genes was found in the same PFGE clusters. The details of these plasmids will be reported elsewhere.

We plan to survey Gram-negative pathogens producing 16S rRNA methylases in 2013 in Vietnam, since more than 9 Gram-negative bacteria producing 16S rRNA methylases have been reported, including *A. baumannii*, *Citrobacter freundii*, *Enterobacter* spp (including *E. cloacae*), *Escherichia coli*, *Klebsiella pneumonia*, *Morganella morganii*, *Proteus mirabilis*, *Providencia* spp (including *P. stuartii*), and *P. aeruginosa*[5].

## Conclusions

This is the first report describing the presence of methylase producing Gram-negative bacteria in medical settings in Southeast Asia, specifically in Vietnam. *A. baumannii* isolates from northern and southern regions of Vietnam may be of different lineages.

## Competing interests

The authors declare that they have no competing interests.

## Authors’ contributions

TT: Performed PCR and sequencing, analyzed data and drafted the manuscript. TMA: Performed MLST analyses. YK, NO, NT and TAT: Performed epidemiological analysis at BMH. NVH, NGB and NQA: Designed protocols and supervised this study at BMH. DMP, TTTN and PHT: Performed clinical bacterial analyses. PTX: Performed epidemiological analysis at CRH. LTAT and NTS: Designed protocols and supervised this study at CRH. TK: Designed protocols and supervised this study. All authors read and approved the final version manuscript.

## Pre-publication history

The pre-publication history for this paper can be accessed here:

http://www.biomedcentral.com/1471-2334/13/251/prepub
